# The perceived impact of an HIV cure by people living with HIV and key populations vulnerable to HIV in the Netherlands: A qualitative study

**DOI:** 10.1016/j.jve.2022.100066

**Published:** 2022-02-25

**Authors:** Kim A.G.J. Romijnders, Laura de Groot, Sigrid C.J.M. Vervoort, Maartje G.J. Basten, Berend J. van Welzen, Mirjam E. Kretzschmar, Peter Reiss, Udi Davidovich, Ganna Rozhnova

**Affiliations:** a Julius Center for Health Sciences and Primary Care, University Medical Center Utrecht, Utrecht University, Utrecht, the Netherlands; b Athena Institute, Vrije Universiteit Amsterdam, the Netherlands; c Department of Innovations in Care, Division Imaging & Oncology, University Medical Center Utrecht, Utrecht, the Netherlands; d Division of Internal Medicine and Dermatology, Department Internal Medicine, University Medical Center Utrecht, Utrecht, the Netherlands; e Department of Internal Medicine, Amsterdam UMC, University of Amsterdam, Amsterdam Infection and Immunity Institute and Amsterdam Public Health Research Institute, Amsterdam, the Netherlands; f Department of Global Health, Amsterdam UMC, University of Amsterdam and Amsterdam Institute for Global Health and Development, Amsterdam, the Netherlands; g Department of Infectious Diseases, Research and Prevention Development, Public Health Service of Amsterdam, Amsterdam, the Netherlands; h Department of Social Psychology, University of Amsterdam, Amsterdam, the Netherlands; i BioISI - Biosystems & Integrative Sciences Institute, Faculdade de Ciências, Universidade de Lisboa, Lisboa, Portugal

**Keywords:** Quality of life, Qualitative research, Sexual and gender minorities, Sexual behaviour, HIV cure, HIV post-treatment control, HIV elimination, ACS, Amsterdam Cohort Studies, ART, Antiretroviral treatment, MSM, Men who have sex with men, PLHIV, People living with HIV, PrEP, Pre-exposure prophylaxis, STI, Sexually transmitted infection, UMCU, University Medical Center Utrecht, QoL, Quality of Life, COREQ, Consolidated criteria for reporting qualitative studies

## Abstract

**Introduction:**

When an HIV cure becomes available, it will have consequences for people living with HIV (PLHIV) and key populations who are vulnerable to HIV. This qualitative study aimed to explore the perceived impact of two HIV cure scenarios (post-treatment control when HIV is suppressed without the need for ongoing antiretroviral treatment (ART) and complete HIV elimination) on the quality of life of PLHIV and key populations living without HIV in the Netherlands.

**Methods:**

Participants were purposefully sampled from the Amsterdam Cohort Studies, the AGEhIV Cohort Study, the outpatient clinic of the University Medical Centre Utrecht and the Dutch HIV Association to increase variability. Semi-structured in-depth interviews were conducted between October 2020 and March 2021 and thematically analysed.

**Results:**

Of the 42 interviewed participants, 29 were PLHIV and 13 represented key populations (i.e., men who have sex with men and people injecting drugs). Both PLHIV and participants from vulnerable key populations hoped that a cure would result in normalization of their lives by removing the need to disclose HIV, reducing stigma and guilt, increasing independence of ART, and liberating sexual behaviour. Both groups believed only HIV elimination could accomplish this desired impact.

**Conclusions:**

While the post-treatment control scenario seems a more plausible outcome of current HIV cure research, our findings highlight that participants may not perceive it as a true cure. Involvement of PLHIV and vulnerable key populations in devising acceptable and feasible experimental approaches to HIV cure is essential to ensure their future successful implementation.

## 1 Introduction

Despite major improvements in disease management, HIV continues to have a significant impact on the quality of life (QoL) of people living with HIV (PLHIV).^1^, ^2^, ^3^ The concept of QoL can be described as an individual's perception of their position in life in relation to their goals, expectations, and standards in the context of a specific culture.^4^ PLHIV and populations who are not living with but are vulnerable to HIV (henceforth, key populations) still experience worse QoL compared to the general population.^2^^,^^5^ Evidence suggests that the complex burden of HIV leads to multiple and profound effects on lives of PLHIV,^3^^,^^5^^,^^6^ highlighting the importance of continuing evaluation and improvement in experienced QoL for PLHIV. For example, de Los Rios and Okoli^1^ demonstrated that the daily use of antiretroviral treatment (ART) is a persistent cause of emotional and physical distress. Another study reported that HIV burden related to medicalization and emotions was found to be transient post HIV diagnosis, while burden related to stigma, feelings of guilt, disclosure, and interpersonal experiences persisted and even increased over time.^3^^,^^7^ Furthermore, among key populations, such as men who have sex with men (MSM), the impact of HIV is considerable due to fear of HIV acquisition and stigmatization of sexual behaviour.^8^^,^^9^

An HIV cure is still hypothetical but its future development is desired and could improve the lives of PLHIV and key populations vulnerable to HIV by removing the experienced or expected burden.^10^, ^11^, ^12^ So far, studies have mainly focused on the willingness of PLHIV to participate in HIV cure research.^13^, ^14^, ^15^, ^16^ Little is known about what an HIV cure would mean to PLHIV or key populations. Few studies addressed how PLHIV perceive a possible cure in addition to willingness to participate in cure research.^8^^,^^17^ They found that participants were willing to partake in a hypothetical HIV cure trial requiring to stop using ART without personal benefits to help others living with HIV.^8^^,^^17^ Power and Dowsett^8^^,^^17^ also found that PLHIV perceived an HIV cure as being free of HIV completely, and that this cure would impact the areas of their life in which HIV is a burden. The differences found between the proposed cure trial, albeit hypothetical, and the desired HIV cure of participants highlight the need to explore how different HIV cure scenarios may impact the lives and QoL of PLHIV. In addition, it is currently unknown whether an HIV cure may result in reduced perception of risk and severity of HIV or reduced preventive behaviours. Understanding the perceptions of PLHIV and vulnerable key populations towards a cure may therefore help guide future public health policy on prevention of HIV and other sexually transmitted infections (STIs). However, to the best of our knowledge, the perceived impact of an HIV cure specifically by key populations has not been studied. The inclusion of key populations, when investigating the expected impact of an HIV cure, is important to direct the research community towards cure interventions that fit the needs of people who are affected by HIV currently and may benefit from a cure in the future. Understanding how a cure might affect the lives of these population groups is key for its successful implementation.^17^^,^^18^

Our qualitative study aims to explore the perceived impact of two different HIV cure scenarios on the lives and QoL of PLHIV and key populations in the Netherlands. In the context of this paper, key populations are people injecting drugs and MSM living without HIV in the Netherlands. We distinguish two hypothetical HIV cure scenarios. The first scenario involves post-treatment control in which a long-term durable viral suppression of HIV is established without the need of ongoing ART, but with a viral reservoir remaining present, potentially allowing for future viral rebound. The second scenario involves complete elimination of HIV from the body.^18^^,^^19^ This cure scenario does not result in immunity to the virus, and cured individuals could hypothetically become re-infected with HIV in future.

## 2 Materials and methods

This qualitative study was approved by the ethics committee of the University Medical Centre Utrecht (UMCU): 20–546/C. Consolidated criteria for reporting qualitative studies (COREQ)^20^ are reported in additional file 1.

### 2.1 Study population

Between October 2020 and March 2021, PLHIV and key populations (i.e., people who inject drugs and MSM) were purposefully sampled from the Amsterdam Cohort Studies (ACS), the AGE_h_IV Cohort Study, the infectious diseases outpatient clinic of the UMCU, and the Dutch HIV Association.^21^ The ACS started the recruitment of MSM in 1984 for an open, ongoing prospective cohort study to investigate the epidemiology, psychosocial determinants, course of infection and pathogenesis of HIV.^22^ The AGE_h_IV cohort recruited PLHIV from the outpatient clinic of the Academic Medical Centre Amsterdam and controls from the Amsterdam Municipal Health Service sexual health clinic and ACS between 2010 and 2012.^23^

Eligibility criteria were being a Dutch or English speaking adult (>18 years), living with HIV, or not having HIV but belonging to key populations. To ensure maximum variation, we included participants who differed by gender, age, sexual orientation, level of education, country of origin, and years since diagnosis for PLHIV. Sampling was scheduled to stop after inductive thematic saturation was reached.^24^

### 2.2 Data collection

Potential participants could contact K.A.G.J.R. to make an appointment for the interview, which was confirmed by e-mail with more information about the study aims and interview procedures. All interviews were audio-recorded and conducted either online using WebEx (n = 31) due to COVID-19 measures or face-to-face at the UMCU (n = 11). Participants received reimbursement for their time (€12,50) and travel expenses.

Semi-structured in-depth interviews were conducted using an interview topic guide (additional file 2). The interviews explored perceptions and motivations in relation to an HIV cure and the perceived impact of two cure scenarios (Table 1). Both the topic guide and the scenarios were pre-tested during a pilot interview.

**Table 1:** Description of cure scenarios.

Type of scenario	Description
Post-treatment control of HIV	Imagine that this treatment has been extensively tested, is safe and available to everyone living with HIV. • This treatment leads to a long-term suppression of HIV by the immune system without the need for ongoing HIV medication. • If HIV is suppressed by the immune system without HIV medication, then: o HIV is still present in the body. o Taking HIV medication is not necessary anymore. o It is not possible to contract HIV again or transfer HIV. o PrEP use or condom use are not necessary to prevent HIV. o There is a small chance that HIV will become active again, that is why it is necessary to have blood tests every six months up till three years after the treatment.
HIV elimination from the body	Imagine that this treatment has been extensively tested, is safe and available to everyone living with HIV. • This treatment removes HIV from the body. • Blood tests every six months up till three years after the treatment are used to determine whether the treatment is successful. • If HIV is successfully removed from the body, then: o It is not necessary to take HIV medication. o There is no chance of HIV becoming active again. o It is not possible to transfer HIV. o It is possible to contract HIV again. This treatment does not provide immunity to HIV. o It is recommended to use PrEP and/or condoms. o It is still advised to get tested for HIV regularly.

The interviews were conducted by K.A.G.J.R., an academic health behavioural scientist. The interview started with an introduction of the study and an explanation of the interview. Next, the interviewer discussed the informed consent orally with the participant and asked whether the participant had any questions. Open-ended questions were used to stimulate participants' own interpretation, and participants were encouraged to elaborately describe their point of view. Prompts were used to further encourage deliberation. In addition, the cure scenarios were discussed to help participants imagine what an HIV cure may look like in future. During the interviews, notes were taken to describe nonverbal communication. Finally, the participants were asked to provide some background information. Interviews lasted from 45 to 90 minutes and were conducted in Dutch (n = 37) or in English (n = 5).

### 2.3 Data analysis

The interviews were conducted in cycles of six.^25^ After each cycle, inductive thematic saturation was assessed, which was reached after the sixth cycle was completed.^24^ The interviews planned for the seventh cycle were still conducted.^25^

Interviews were transcribed verbatim. Data were systematically analysed by K.A.G.J.R. and L.d.G. according to the thematic analysis of Braun and Clarke^26^ to reflect a greater degree of data transformation.^27^ We aimed for interpretative thematic analysis to go beyond a descriptive level, to identify the underpinning explanation of the obtained data.^26^^,^^27^ Both researchers coded the data independently and discussed afterwards to reach consensus. The analysis was also discussed as part of a peer review with an expert in qualitative research (S.C.J.M.V.).^26^ The main analysis consisted of six stages^26^ (Table 2), enabling us to identify recurring topics, ideas and patterns within the data. Data analysis was supported by NVivo 12.^28^ Dutch quotes were translated into English by K.A.G.J.R. and L.d.G. using the forward-backward method. To enhance the reliability of the data analysis, we used the 15-item checklist by Braun and Clarke^26^ (additional file 3).

**Table 2:** Stages of thematic analysis.

Stage	Description
1) Familiarization of data	Data were transcribed, transcripts were re-read and initial ideas were noted down by K.A.G.J.R. and L.d.G.
2) Generating initial codes	Data was coded by K.A.G.J.R. and L.d.G. in a systematic fashion across the entire data set by using NVivo. Matching data relevant for codes were automatically grouped.
3) Searching for themes	Different codes were matched by K.A.G.J.R. and L.d.G. in potential themes, so that all relevant data were coded and gathered amongst these potential themes. Subsequently, K.A.G.J.R. and L.d.G. looked for more patterns or repetition discerned in the data, which allowed us to move away from the topics raised. In this way, a thematic map was generated.
4) Reviewing themes	Coded extracts for the themes discerned were reviewed by K.A.G.J.R. and L.d.G. to search for coherent patterns. After the initial review of coded extracts, transcripts were re-read to consider the validity of the themes. By reviewing the coded extracts and transcripts for the themes discerned, it was checked whether the meaning of the themes represented the data.
5) Defining and naming themes	Specifics of each theme were continuously refined by K.A.G.J.R. and L.d.G. to make sure that the overall story of the data was conveyed by the defined themes. Thereafter, clear ideas and concepts were generated for each theme. These themes were peer reviewed by a qualitative research expert (S.C.J.M.V.).
6) Producing the report	Vivid compelling examples – quotes directly deriving from our data – were provided by K.A.G.J.R. and L.d.G. related to the themes and our main research question, ultimately leading to the report of our study.

## 3 Results

### 3.1 Participant characteristics

Forty-four participants scheduled an interview, of whom 42 eventually participated in the study. The reasons for not participating in the study could not be obtained. Nine of the 42 participants were women, one was a transwoman, 29 were living with HIV, and 13 belonged to key populations. Age ranged between 24 and 72 years. Twenty-six participants were gay, 12 heterosexual, and four bisexual. Twenty-three participants were born in the Netherlands, two in Italy, one in the United States of America, Russia, the United Kingdom, Germany, South Africa, and Tanzania. Participants' characteristics are described in Table 3.

**Table 3:** Characteristics of participants.

	PLHIV (n, %)	Key populations (n, %)
N	29	13
Mean age in years [range]	46 [27–72]	48 [24–72]
**Education**^a^		
Low	0	1 (8%)
Middle	6 (21%)	0
High	23 (79%)	12 (92%)
**Gender**		
Male	19 (66%)	13 (100%)
Female^b^	10 (34%)	0
**Sexual orientation**		
Gay	14 (48%)	12 (92%)
Heterosexual	11 (38%)	1 (8%)
Bisexual	4 (14%)	0
**Migration background**		
Yes	6 (21%)	2 (15%)
No	23 (79%)	11 (85%)
**Living with HIV in years**		
Less than 1 year	2 (7%)	
1–5 years	7 (24%)	
5–10 years	7 (24%)	
10–15 years	3 (10%)	
15–20 years	4 (14%)	
20 years or more	6 (21%)	

a Low: primary and lower secondary, middle: upper secondary and post-secondary, and high: bachelor, master, doctoral or equivalent.

b One of the female participants was a transwoman.

### 3.2 What an HIV cure would mean to participants

Perceptions of PLHIV and individuals without HIV from key populations did not seem to differ much in their perception of an HIV cure. Many participants expressed that they knew little about the current developments in the field of HIV cure, but there was consensus on what an HIV cure would mean to them. For participants, a treatment can be considered an HIV cure if it ensures that HIV is completely removed from the body, if there is no chance of rebound of viral load, and if there is no risk of transmitting HIV anymore. This description was in line with the HIV elimination scenario. Post-treatment control was viewed by participants as the next step in HIV treatment, but not as a cure.

What I mean by an HIV cure is that there are pills that ensure that the virus is gone from the body for good. And when you are cured then you can continue medicine free through life … that's what I hope it [HIV cure] is. [male, 60 year old, gay, living with HIV for 15 years).

It's great that I wouldn't have to take medication anymore [after post-treatment control], but you'll never know for sure it won't come back. With the second scenario [HIV elimination] you know it won't come back. Then … you'll feel truly cured. [male, 60 year old, gay]

### 3.3 Hoping for a future without HIV

Several perceptions regarding HIV cure were reported by participants. Some PLHIV hoped that a cure would become reality in their lifetime. These participants described a cure as a possibility to look forward to a future without HIV. The perceptions of many participants belonging to key populations living without HIV agreed with those of PLHIV in their hope that an HIV cure would become a reality.

If it [HIV] could be cured, that would be amazing. Count me in! I'll be the first in line, really! I really want it because it's so inconvenient now … [female, 39 year old, heterosexual, living with HIV for 10 years]

Other PLHIV were able to accept living with HIV and they were neither hopeful nor pessimistic about an HIV cure. They seemed to want to manage their expectations and protect themselves against potential disappointment.

I hope that they succeed … maybe I won't live to see it [an HIV cure] … and we shouldn't forget that what we would want and what we would get may not be the same. [male, 50 year old, gay, living with HIV for 2 years]

Finally, there were also PLHIV who currently were struggling with living with HIV and they seemed unable to perceive a cure and its plausibility.

I feel it won't be realistic in my own lifetime … I don't really put my focus or my hopes in that. My focus is really the daily struggles with the health condition … Like I feel it's [a cure is] sort of speculation. [female, 42 year old, heterosexual, living with HIV for 6 years]

### 3.4 An HIV cure would normalize my life

Both PLHIV and interviewees without HIV described to strive for normality in their current life despite HIV . When discussing the perceived impact of a cure, participants mentioned they did not think it would have a major impact on their life. When prompted further, PLHIV mentioned that HIV elimination would be the only way for them to have a fresh start and be like everybody else again.

[about HIV elimination] For sure, yes [it's a cure]. Then you're just like everybody else. [female, 59 year old, heterosexual, living with HIV for 36 years]

Currently, HIV overshadowed lives of participants due to having to disclose their HIV status, feeling a lack of control in their life because of their dependency on medication, feelings of guilt about acquiring HIV, and feeling restricted in their sexual behaviour.

Physically it's nothing, mentally on the other hand [having HIV] is like being a closeted gay man … It is like you always have a secret … [my life] is no longer as carefree as before I had HIV. I didn't have that extra shadow that I have now … mentally I have this extra thing, that shadow. And it [HIV elimination] would mean that this shadow that is always with me would disappear … The closet would be gone! [male, 42 year old, gay, living with HIV for 3 years]

PLHIV believed that post-treatment control of HIV would not have the same effect because HIV would still be present in the body, and they felt the shadow of HIV would remain. Similarly, interviewees from key populations considered that only HIV elimination would make them feel truly cured and enable them to live normal lives.

[About HIV post-treatment control] You still need that control. And that virus is still in your body … So let me put it this way, I won't feel cured then. [female, 53 year old, heterosexual, living with HIV for 9 years]

The following sub-themes explain in detail how a cure would impact the aspects of life HIV currently overshadows.

#### 3.4.1 Elimination of HIV would mean I don't have to disclose anymore

Participants mentioned that only HIV elimination would make them feel cured because there would be no need to disclose. PLHIV explained that a large part of the overshadowing presence of HIV was disclosing, which was considered very personal, dependent on the situation, and particularly cognitively challenging.

It's [HIV] still a secret, so it would be great for me if I didn't have this secret anymore … Well, this [elimination of HIV] would be fantastic! I wouldn't have to take any medication anymore; I would feel really cured … You'll be just like everybody else who isn't living with HIV. You don't have anything, so you don't have hide anything … [female, 53 year old, heterosexual, living with HIV for 9 years]

Interviewees from both groups could easily imagine that – based on experiences – disclosing you are living with HIV is or would be considered very difficult. They believed HIV elimination could solve this.

I have a secret; I choose not to share this [HIV] with everyone … It's [life with HIV] a bit of a double life … a life with a secret. And the feeling it gives is to be different and that often brings a bit of loneliness … It will never go away, so it's also a piece of powerlessness. Yes, life has changed forever and … That often still brings frustration … sometimes also anger, but above all, a lot of sadness. If I could be cured [HIV elimination], I'd jump at that chance. [male, 38 year old, bisexual, living with HIV for 6 years]

Participants from both groups believed HIV post-treatment control could not have the same effect as HIV elimination because there would still be a need to disclose HIV.

Well, because then [after elimination of HIV] you don't have to explain everything to someone every time. Look, it's much more fun when I'm on a date with you that I can talk about your work and about your parents and how your childhood was. Instead of having to tell you at some point that I have a chronic illness. [male, 33 year old, heterosexual, living with HIV for three months]

#### 3.4.2 Elimination of HIV would reduce my feelings of guilt

Both PLHIV and individuals from key populations hoped that elimination of HIV may transform the perception of the public of HIV from an incurable illness to a curable STI, and, as a result, reduce the currently experienced stigmatization and guilt for acquiring HIV.

I feel ashamed … and guilty about the way I contracted it [HIV]. Yeah, I really want to right this [HIV] wrong. That's my hope … and it [elimination of HIV] provides perspective. Then I can say “I don't have it [HIV] anymore, its gone! I've righted this wrong, I would be so lucky! I don't have to be ashamed anymore. [male, 45 year old, gay, living with HIV for 11 years]

These feelings of guilt seemed to increase if the social environment wondered whose fault having HIV was. MSM without HIV explained that they feared the guilt of contracting HIV especially now with all the available knowledge and resources, such as PrEP, to prevent HIV.

Everybody wants to know how you got it [HIV]. That's such a weird question because if somebody has long cancer or diabetes, would you ask: “How did you get it?” If you think about this, then I think about ‘‘Whose fault is it?” [female, 39 year old, heterosexual, living with HIV for 10 years]

The taboo would be that I would have contracted it, so there is a certain irresponsibility in it. … So, it would be a certain … A loss of face because of an irresponsibility that I would have contracted it [HIV] with the resources [e.g., PrEP] that we have. [male, 33 year old, gay]

PLHIV hoped that the feelings of guilt would disappear after HIV elimination becomes reality, in a similar way that they did not feel guilty for acquiring other STIs.

I think that the taboo on HIV would disappear the moment it is no longer a chronic illness, but a normal STI. For which you can just take a simple pill and you are rid of it [HIV]. [male, 27 year old, gay, living with HIV for 3 years]

In the case of the post-treatment control scenario, both PLHIV and participants without HIV believed that stigmatization would not change. In addition, participants shared the concern that feelings of guilt and experienced stigmatization would depend on the type and intensity of an HIV cure that would become available. MSM without HIV feared that the stigmatization after HIV elimination would be worse if someone would contract HIV or – even worse – someone would contract HIV again. In addition, some participants expressed fear that an HIV cure would only work once and not after re-infection, and thereby stressed the importance of responsible sexual behaviour after being cured.

It's not that it's one pill [about a cure] and you're done … somebody would have to follow the treatment again. I can only assume that people would talk about that behind their backs … that they have an opinion about it … and disapprove. YES [about thinking there would be more stigmatization]! [male, 56 yearold, gay]

#### 3.4.3 An HIV cure would provide me with more control over my life

Participants from both groups believed that both post-treatment control and HIV elimination would increase their ability to take charge of their own life because there would be no need to take ART. On one hand, the idea of having to take ART for the rest of their lives and its side effects were mentioned as aspects of HIV negatively influencing the ability of participants to take charge of their life. PLHIV and key populations hoped that both scenarios could provide a new sense of freedom in organizing and planning the life of PLHIV.

I just schedule my appointments at work to make sure I always eat and take my pills at the same time. Look, you wouldn't have to do that kind of thing anymore [after post-treatment control. [female, 43 year old, heterosexual, living with HIV for 15 years]

On the other hand, PLHIV experienced a sense of control with ART. If post-treatment control would become available, some PLHIV felt they would need to let go of this certainty they now experienced with regards to ART and HIV, for example:

So that [post-treatment control] would take me back to a less spontaneous form of sex where I'm going to use a condom because I don't want to take the risk that he [partner] might get infected. But life without medication … That is of course very attractive, mmm … [male, 31 year old, gay, living with HIV for 5 years]

#### 3.4.4 An HIV cure would help me not to feel restricted in my sexual freedom

PLHIV and key populations hoped that both post-treatment control and HIV elimination may change their experienced sexual freedom by reducing fear of HIV and changing the definition of safer and unsafe sex. Participants speculated that an HIV cure would increase the feeling of sexual freedom to levels pre-dating HIV/AIDS because the fear of HIV would disappear.

Then you get back to the sexual freedom that was evidently there before there was HIV/AIDS [about a cure]. You always must take other STIs into account, but the worst would disappear. [male, 55 year old, gay]

In addition, before the era of HIV/AIDS, safer sex meant preventing unwanted pregnancy and that the definition of safer sex as we know it today emerged after the beginning of the HIV epidemic. This definition was strongly related to experiences in sexual freedom.

I was of the generation that we had the pill [birth control] and so you could grow in your sexuality for the first time because you weren't afraid anymore [of pregnancy] … and HIV wasn't there yet. At least, we didn't know about that yet … [female, 58 year old, heterosexual, living with HIV for 32 years]

In addition, MSM cautioned that an HIV cure and a perceived increase in sexual freedom would not mean the absence of risk of other STIs, and they expressed some concern that HIV elimination might not work again after one would re-acquire HIV.

If you have contracted HIV, it requires, by definition, some kind of behavioural change. I know a number of people who have HIV. They became very reckless afterwards because they believed it didn't matter anymore. The worst that could happen, “I may get HIV”, has already happened … When you're cured [HIV elimination], you'll have to change again … There will certainly be a group of people who find that difficult, and then suppressing HIV [post-treatment control] may be an option for them. With a cure [elimination of HIV] you really have to make sure that a lifestyle changes too, even though that can sometimes be very condescending. [male, 30 year old, gay]

A hope was expressed that an HIV cure would provide a new definition of safer sex, and, as a result, it would change the experienced sexual freedom.

[about sexual behaviour change and freedom] It's about the image [of HIV], with both [cure] scenarios it changes to a STI. You already see that due to PrEP, condom use reduced among gay men, I think if there is a cure for HIV and you fast forward 20 years, then you probably wouldn't use either [PrEP or condoms] anymore. [male, 33 year old, gay]

## 4 Discussion

Our thematic analysis demonstrated the perceptions concerning the impact of an HIV cure for PLHIV and individuals belonging to key populations in the Netherlands. The most desired aspect was the elimination of the multifaceted and overshadowing presence of HIV, that would result in normalization of participants' lives, by removing the need to disclose HIV, reducing stigma and guilt, increasing independency of ART, and liberating sexual behaviour. Both groups believed only HIV elimination could accomplish this desired impact. The similarity in perceptions about the impact of an HIV cure among PLHIV and key populations may be explained by our homogeneous participant sample of key populations, of which many were MSM. Participating MSM without HIV reported to know MSM with HIV. They seemed to fear and be able to imagine the overshadowing presence of HIV in their lives and therefore perceive the impact of an HIV cure.^29^ This may explain why they had a clear idea about the impact of a cure and why their perceptions about this impact were relatively similar to those of PLHIV.

Both PLHIV and key populations perceived the ultimate effect of an HIV cure to be the normalization of life. However, when asked about the potential impact of a cure, participants started out by explaining that they believed not to be major. This may be explained by their current striving for normalization of HIV in day-to-day life and may be due to habituation.^3^^,^^29^^,^^30^ In addition, this may indicate that PLHIV do feel satisfied about their life of which HIV became a part and could make people perceive less of an impact of an HIV cure on QoL.^12^ An HIV cure may still prove vital in improving the QoL of PLHIV and key populations.

The sub-theme “elimination of HIV would mean I don't have to disclose anymore” revealed that a large part of being just like everybody else again was not having to disclose one's HIV status. Disclosing or deciding to keep HIV a secret was considered one of the most burdensome aspects of HIV^29^^,^^31^^,^^32^ and PLHIV often experienced stigmatization during interpersonal disclosure interactions. They hoped normalization after HIV elimination would mean the disappearance of the need for such interactions, and thus, stigmatization. Feelings of guilt were linked to the experienced stigmatization during interpersonal interactions about HIV. Hence, both PLHIV and those without HIV hoped that HIV elimination may reduce feelings of guilt surrounding HIV acquisition. However, they also worried guilt may increase. Compared to other chronic diseases, HIV – and, for example, obesity or hepatitis C among MSM – evoke less sympathy from society because people hold those with the disease responsible for their (sexual) behaviour.^33^, ^34^, ^35^, ^36^ Consequently, it can be hypothesized that there would be even less sympathy from society if one would contract HIV – again – after its elimination,especially, if HIV elimination was time-consuming, costly, and intensive; it ultimately may result in more stigmatization, and thus, difficulty with disclosure and feelings of guilt.

Previous research showed that the clinical burden of ART was quickly accustomed to and perceived as acceptable over time by PLHIV,^3^^,^^7^^,^^37^, ^38^, ^39^, ^40^ yet our results demonstrated that ART was considered burdensome due to a lack of control by PLHIV and key populations. Participants reported that a cure would provide participants with more control and flexibility in their lives because they would no longer be dependent on ART. Even though research suggested that PLHIV usually accustom to daily ART, it may continuously remind people of having HIV.^12^ Both HIV post-treatment control and its elimination may resolve this issue since ART would no longer be needed.

Lastly, participants thought a cure would provide more sexual freedom. First, PLHIV and key populations were hoping that a cure would lead to sex without fear of transmitting HIV. Previous research demonstrated that the fear of transmitting HIV to a partner often remains burdensome for PLHIV.^41^^,^^42^ For PLHIV and key populations, this fear about onward transmission only seems to disappear in the case of HIV elimination. Moreover, HIV elimination may provide more freedom in the future development of MSM's sexuality. In addition, PLHIV and key populations hoped that categorizing sex into safer and unsafe^43^, ^44^, ^45^, ^46^ would no longer be needed after a cure was available. The emergence of safer sex norms seems to have contributed to stigmatization and feelings of guilt with regards to condomless sexual behaviour, whether it led to contracting HIV or not.

This study has several strengths. While previous studies have focused mainly on exploring the willingness of PLHIV to participate in HIV cure related trials,^13^, ^14^, ^15^, ^16^ our study explored the perceived impact of an HIV cure among both PLHIV and key populations. Our results highlight the wishes with regards to an HIV cure of both key potential end-users of a cure. Involvement of PLHIV and key populations in HIV cure research is essential to ensure that their expectations, ideas, and opinions are considered in the design of possible future interventions aimed at achieving a cure.^14^^,^^18^^,^^47^, ^48^, ^49^ Although our results showed similarities between the perceptions of the impact of cure scenarios among PLHIV and key populations, differences in perceptions may exist in other key populations, such as sex workers and should be addressed by further research. Finally, reliability of the data was enhanced by using the validated Braun and Clark checklist and the COREQ.^20^ This study also has some limitations. Despite variation in the country of origin, all study participants currently lived in the Netherlands, indicating accessibility to ART. Additional research should explore the perceived impact an HIV cure may have in areas without universal access to ART. Selection may have occurred towards highly educated persons.

## 5 Conclusions

In conclusion, according to study participants elimination of HIV would remove its multifaceted and overshadowing presence in the lives of PLHIV and key populations. While post-treatment control of HIV in the short-term may be a more plausible outcome of research towards an HIV cure, individuals may not perceive this as a true cure. Involvement of PLHIV and key populations in devising acceptable and feasible experimental approaches to HIV cure is essential to ensure their future successful acceptability and implementation.

## Authors' contributions

Kim A.G.J. Romijnders: Conceptualization; Data curation; Formal analysis; Investigation; Methodology; Project administration; Resources; Supervision; Validation; Writing - original draft; Writing - review & editing.

Laura de Groot: Data curation; Formal analysis; Methodology; Validation; Writing - review & editing.

Sigrid C.J.M. Vervoort: Formal analysis; Methodology; Validation; Writing - review & editing.

Maartje G.J. Basten: Conceptualization; Funding acquisition; Writing - review & editing.

Berend J. van Welzen: Investigation; Resources; Writing - review & editing.

Mirjam E. Kretzschmar: Methodology; Writing - review & editing.

Peter Reiss: Methodology; Writing - review & editing.

Udi Davidovich: Methodology; Writing - review & editing.

Ganna Rohznova: Conceptualization; Funding acquisition; Supervision; Investigation; Project administration; Resources; Writing - review & editing.

## Funding

The authors gratefully acknowledge funding by the Aidsfonds Netherlands, grant number P-52901.

## Declaration of competing interest

The authors declare that they have no known competing financial interests or personal relationships that could have appeared to influence the work reported in this paper.
