# Supplementary material for: The perceived impact of an HIV cure by people living with HIV and key populations vulnerable to HIV in the Netherlands: A qualitative study
Source: J Virus Erad. 2022 Feb 25;8(1):100066. doi: 10.1016/j.jve.2022.100066 (PMC8907669; doi:10.1016/j.jve.2022.100066)
Supplement: Multimedia component 1 [file mmc1.pdf]

Additional file 1: COREQ

The perceived impact of an HIV cure by people living with HIV and key populations vulnerable to HIV in the Netherlands: A qualitative study

Kim A.G.J. Romijnders [a,*], Laura de Groot [a,b,1], Sigrid C.J.M. Vervoort [c,1], Maartje G.J. Basten [a], Berend J. van Welzen [d], Mirjam E. Kretzschmar [a], Peter Reiss [e,f], Udi Davidovich [g,h], Ganna Rozhnova [a,i]

a Julius Center for Health Sciences and Primary Care, University Medical Center Utrecht, Utrecht University, Utrecht, the Netherlands

b Athena Institute, Vrije Universiteit Amsterdam, the Netherlands

c Department of Innovations in Care, Division Imaging & Oncology, University Medical Center Utrecht, Utrecht, the Netherlands

d Division of Internal Medicine and Dermatology, Department Internal Medicine, University Medical Center Utrecht, Utrecht, the Netherlands

e Department of Internal Medicine, Amsterdam UMC, University of Amsterdam, Amsterdam Infection and Immunity Institute and Amsterdam Public Health Research Institute, Amsterdam, the Netherlands

f Department of Global Health, Amsterdam UMC, University of Amsterdam and Amsterdam Institute for Global Health and Development, Amsterdam, the Netherlands

g Department of Infectious Diseases, Research and Prevention Development, Public Health Service of Amsterdam, Amsterdam, the Netherlands

h Department of Social Psychology, University of Amsterdam, Amsterdam, the Netherlands

i BioISI - Biosystems & Integrative Sciences Institute, Faculdade de Ciˆencias, Universidade de Lisboa, Lisboa, Portugal

* Corresponding author. Universiteitsweg 100, Utrecht, 3584, CG, the Netherlands.

E-mail addresses: k.a.g.romijnders@umcutrecht.nl (K.A.G.J. Romijnders), l.de.groot@vu.nl (L. de Groot), s.vervoort@umcutrecht.nl (S.C.J.M. Vervoort), M.G.J. Basten-3@umcutrecht.nl (M.G.J. Basten), b.j.vanwelzen@umcutrecht.nl (B.J. van Welzen), m.e.e.kretzschmar@umcutrecht.nl (M.E. Kretzschmar), p.reiss@ amsterdamumc.nl (P. Reiss), udavidovich@ggd.amsterdam.nl (U. Davidovich), G.Rozhnova@umcutrecht.nl (G. Rozhnova). 1 These authors have contributed equally to this work.

Consolidated Criteria for Reporting Qualitative Studies

Domain 1: Research team and reflexivity		Pages
Personal Characteristics		
1. Interviewer/facilitator	Which author/s conducted the interview or focus group? K.A.G.J.R. conducted all the interviews.	6
2. Credentials	PhD	n/a
3. Occupation	Assistant Professor	n/a
4. Gender	Female	n/a
5. Experience and training	Extensive training and over 5 years of experience in qualitative research.	6
Relationship with participants		
6. Relationship established	No	6
7. Participant knowledge of the interviewer	The participants received an information flyer prior to the interview and a letter of invitation after applying for an interview. For most interviewees, this study topic was of personal interest.<br><br>The researcher had no personal goals with regards to this study, other than to complete the aims and objectives of the study. As part of the informed consent procedure, the participants were fully informed about the aims and the objectives of the study.	6
8. Interviewer characteristics	What characteristics were reported about the interviewer/facilitator? e.g., Bias, assumptions, reasons, and interest in the research topic<br><br>The main researcher is a social scientist with extensive qualitative research experience and no medical background.	6
Domain 2: study design		
Theoretical framework		

9. Methodological orientation and Theory	What methodological orientation was stated to underpin the study? e.g., grounded theory, discourse analysis, ethnography, phenomenology, content analysis<br><br>A thematical analysis was performed [31, 32].	7-8
Participant selection		
10. Sampling	How were participants selected? e.g., purposive, convenience, consecutive, snowball<br><br>Participants were purposely sampled with the help of the partners at the Amsterdam Cohort Studies, the AGE$_h$IV Cohort Study, the infectious diseases outpatient clinic of the UMCU, and the Dutch HIV Association	5-6
11. Method of approach	How were participants approached? e.g., face-to-face, telephone, mail, email<br><br>Participants who showed interest in being interviewed were contacted by telephone by K.A.G.J.R. and received a follow-up email with the confirmation of the date and time, and the reminder a day before the interview.	5-6
12. Sample size	How many participants were in the study?<br>42	5-9
13. non-participation	How many people refused to participate or dropped out? Reasons?<br>Of those who showed interested in the study, 2 dropped out.	9
Setting		
14. Setting of data collection	Where was the data collected? e.g., home, clinic, workplace<br><br>Due to Covid-19 restrictions, 31 interviews were conducted online using WebEx meetings and 11 were conducted face-to-face at the UMCU	6
15. Presence of non-participants	Was anyone else present besides the participants and researchers?<br>No.	n/a

16. Description of sample	What are the important characteristics of the sample? e.g., demographic data, date<br>44 participants signed up, of whom 42 eventually participated in the study. The reasons for not participating in the study could not be obtained. Ten of the 42 participants were women, one of whom was a transwoman, 29 were living with HIV, and 13 belonged to key populations. Age ranged between 24 and 72 years. 26 participants considered themselves as gay, 12 as straight, and four as bisexual. 23 participants were born in the Netherlands, two in Italy, one in the United States of America, Russia, the United Kingdom, Germany, South Africa, and Tanzania.	9
Data collection		
17. Interview guide	Were questions, prompts, guides provided by the authors? Was it pilot tested?<br>Semi-structured in-depth interviews were conducted using an interview topic guide (additional file 2). The interviews explored perceptions, experiences, feelings, and motivations in relation to an HIV cure and the perceived impact of two cure scenarios (table 1). Both the topic guide and the scenarios were pre-tested during a pilot interview. The topic guide was used as a flexible tool to allow in-depth exploration of emergent themes, topics, and ideas.	6, 26
18. Repeat interviews	Were repeat interviews carried out? If yes, how many?<br><br>No.	n/a
19. Audio/visual recording	Did the research use audio or visual recording to collect the data?<br><br>All interviews were audio recorded.	6-8
20. Field notes	Were field notes made during and/or after the interview or focus group?<br><br>During the interviews, notes were taken to describe nonverbal communication, such as bodily movements and facial expressions.	6-8

21. Duration	What was the duration of the interviews or focus group?<br><br>60 to 90 minutes.	6-8
22. Data saturation	Was data saturation discussed?<br>The interviews were conducted in cycles of six[30]. After each cycle, inductive thematic saturation was assessed, which was reached after the sixth cycle was completed[29]. The interviews planned for the seventh cycle were still conducted[30].	6-8
23. Transcripts returned	Were transcripts returned to participants for comment and/or correction?<br><br>No transcripts were not returned to participants.	n/a
Domain 3: analysis and findings		
Data analysis		
24. Number of data coders	How many data coders coded the data?<br><br>Data were systematically analysed by K.A.G.J.R. and L.D.G according to the thematic analysis of [31] to reflect a greater degree of data transformation [32]. We aimed for interpretative thematic analysis to go beyond a descriptive level, to identify the underpinning explanation of the obtained data [31, 32]. Both researchers coded the data independently and discussed afterwards to reach consensus. The analysis was also discussed as part of a peer review with an expert in qualitative research (S.C.J.M.V.)[31].	7-9
25. Description of the coding tree	Did authors provide a description of the coding tree?<br><br>No.	n/a

26. Derivation of themes	Were themes identified in advance or derived from the data?<br><br>Data were systematically analysed by K.A.G.J.R. and L.D.G according to the thematic analysis of [31] to reflect a greater degree of data transformation [32]. We aimed for interpretative thematic analysis to go beyond a descriptive level, to identify the underpinning explanation of the obtained data [31, 32]. No themes were identified in advance.	7-9
27. Software	What software, if applicable, was used to manage the data?<br><br>NVivo version 12 was used to manage the data.	8
28. Participant checking	Did participants provide feedback on the findings?<br><br>No	n/a
Reporting		
29. Quotations presented	Were participant quotations presented to illustrate the themes / findings? Was each quotation identified? e.g., participant number<br><br>Yes, quotations are used to illustrate themes found. No identifier was used to protect the privacy of participants.	9-16
30. Data and findings consistent	Was there consistency between the data presented and the findings?<br><br>Yes.	9-16
31. Clarity of major themes	Were major themes clearly presented in the findings?<br><br>Yes.	9-16
32. Clarity of minor themes	Is there a description of diverse cases or discussion of minor themes?<br><br>Themes and sub-themes are discussed in the results.	9-16
