# Supplementary material for: The perceived impact of an HIV cure by people living with HIV and key populations vulnerable to HIV in the Netherlands: A qualitative study
Source: J Virus Erad. 2022 Feb 25;8(1):100066. doi: 10.1016/j.jve.2022.100066 (PMC8907669; doi:10.1016/j.jve.2022.100066)
Supplement: Multimedia component 2 [file mmc2.pdf]

Additional file 2: Interview topic guide

The perceived impact of an HIV cure by people living with HIV and key populations vulnerable to HIV in the Netherlands: A qualitative study

Kim A.G.J. Romijnders [a,*], Laura de Groot [a,b,1], Sigrid C.J.M. Vervoort [c,1], Maartje G.J. Basten [a], Berend J. van Welzen [d], Mirjam E. Kretzschmar [a], Peter Reiss [e,f], Udi Davidovich [g,h], Ganna Rozhnova [a,i]

a Julius Center for Health Sciences and Primary Care, University Medical Center Utrecht, Utrecht University, Utrecht, the Netherlands

b Athena Institute, Vrije Universiteit Amsterdam, the Netherlands

c Department of Innovations in Care, Division Imaging & Oncology, University Medical Center Utrecht, Utrecht, the Netherlands

d Division of Internal Medicine and Dermatology, Department Internal Medicine, University Medical Center Utrecht, Utrecht, the Netherlands

e Department of Internal Medicine, Amsterdam UMC, University of Amsterdam, Amsterdam Infection and Immunity Institute and Amsterdam Public Health Research Institute, Amsterdam, the Netherlands

f Department of Global Health, Amsterdam UMC, University of Amsterdam and Amsterdam Institute for Global Health and Development, Amsterdam, the Netherlands

g Department of Infectious Diseases, Research and Prevention Development, Public Health Service of Amsterdam, Amsterdam, the Netherlands

h Department of Social Psychology, University of Amsterdam, Amsterdam, the Netherlands

i BioISI - Biosystems & Integrative Sciences Institute, Faculdade de Ciˆencias, Universidade de Lisboa, Lisboa, Portugal

* Corresponding author. Universiteitsweg 100, Utrecht, 3584, CG, the Netherlands.

E-mail addresses: k.a.g.romijnders@umcutrecht.nl (K.A.G.J. Romijnders), l.de.groot@vu.nl (L. de Groot), s.vervoort@umcutrecht.nl (S.C.J.M. Vervoort), M.G.J. Basten-3@umcutrecht.nl (M.G.J. Basten), b.j.vanwelzen@umcutrecht.nl (B.J. van Welzen), m.e.e.kretzschmar@umcutrecht.nl (M.E. Kretzschmar), p.reiss@ amsterdamumc.nl (P. Reiss), udavidovich@ggd.amsterdam.nl (U. Davidovich), G.Rozhnova@umcutrecht.nl (G. Rozhnova). 1 These authors have contributed equally to this work.

Interview topic guide

Topic	Example questions or description
**Introduction interview**	Introduction of the interview by the interviewer.
**Informed consent**	The participant provided written or verbal informed consent.
**Current situation of the participant**	
Daily life with HIV	"What is the role of HIV in your life?"<br>"Do you remember what you thought of HIV, before you had HIV?"
Quality of life	"What provides you with quality in life?"<br>"What does quality of life mean to you?"<br>"Could you explain why it provides you quality?"
Sexual freedom	"How would you describe your sexual freedom?"
**An HIV cure**	
Perceptions of an HIV cure	"What does an HIV cure mean to you?"<br>"What would cure treatment look like?"<br>"Is it something you would be interested in?"
Impact of an HIV cure: post-treatment control<br><br>Impact of an HIV cure: HIV elimination	"What is the first thing that comes to mind when you hear about this scenario?"<br>"You mentioned XXX about quality in life…How would they change if this scenario would become a reality?"<br>"What would this type of treatment mean to you?"
**Closing**	Closing of the interview.
