# Supplementary material for: The perceived impact of an HIV cure by people living with HIV and key populations vulnerable to HIV in the Netherlands: A qualitative study
Source: J Virus Erad. 2022 Feb 25;8(1):100066. doi: 10.1016/j.jve.2022.100066 (PMC8907669; doi:10.1016/j.jve.2022.100066)
Supplement: Multimedia component 3 [file mmc3.pdf]

Additional file 3: 15-item checklist by Braun and Clarke [31]

The perceived impact of an HIV cure by people living with HIV and key populations vulnerable to HIV in the Netherlands: A qualitative study

Kim A.G.J. Romijnders [a,*], Laura de Groot [a,b,1], Sigrid C.J.M. Vervoort [c,1], Maartje G.J. Basten [a], Berend J. van Welzen [d], Mirjam E. Kretzschmar [a], Peter Reiss [e,f], Udi Davidovich [g,h], Ganna Rozhnova [a,i]

a Julius Center for Health Sciences and Primary Care, University Medical Center Utrecht, Utrecht University, Utrecht, the Netherlands

b Athena Institute, Vrije Universiteit Amsterdam, the Netherlands

c Department of Innovations in Care, Division Imaging & Oncology, University Medical Center Utrecht, Utrecht, the Netherlands

d Division of Internal Medicine and Dermatology, Department Internal Medicine, University Medical Center Utrecht, Utrecht, the Netherlands

e Department of Internal Medicine, Amsterdam UMC, University of Amsterdam, Amsterdam Infection and Immunity Institute and Amsterdam Public Health Research Institute, Amsterdam, the Netherlands

f Department of Global Health, Amsterdam UMC, University of Amsterdam and Amsterdam Institute for Global Health and Development, Amsterdam, the Netherlands

g Department of Infectious Diseases, Research and Prevention Development, Public Health Service of Amsterdam, Amsterdam, the Netherlands

h Department of Social Psychology, University of Amsterdam, Amsterdam, the Netherlands

i BioISI - Biosystems & Integrative Sciences Institute, Faculdade de Ciˆencias, Universidade de Lisboa, Lisboa, Portugal

* Corresponding author. Universiteitsweg 100, Utrecht, 3584, CG, the Netherlands.

E-mail addresses: k.a.g.romijnders@umcutrecht.nl (K.A.G.J. Romijnders), l.de.groot@vu.nl (L. de Groot), s.vervoort@umcutrecht.nl (S.C.J.M. Vervoort), M.G.J. Basten-3@umcutrecht.nl (M.G.J. Basten), b.j.vanwelzen@umcutrecht.nl (B.J. van Welzen), m.e.e.kretzschmar@umcutrecht.nl (M.E. Kretzschmar), p.reiss@ amsterdamumc.nl (P. Reiss), udavidovich@ggd.amsterdam.nl (U. Davidovich), G.Rozhnova@umcutrecht.nl (G. Rozhnova). 1 These authors have contributed equally to this work.

15-item checklist by Braun and Clarke [31]

Process	No.	Criteria
**Transcription**	1	The data have been transcribed to an appropriate level of detail, and the transcripts have been checked against the tapes for 'accuracy'.
**Coding**	2	Each data item has been given equal attention in the coding process.
	3	Themes have not been generated from a few vivid examples (an anecdotal approach), but instead the coding process has been thorough, inclusive, and comprehensive.
	4	All relevant extracts for all each theme have been collated.
	5	Themes have been checked against each other and back to the original data set.
	6	Themes are internally coherent, consistent, and distinctive.
**Analysis**	7	Data have been analysed – interpreted, made sense of – rather than just paraphrased or described.
	8	Analysis and data match each other – the extracts illustrate the analytic claims.
	9	Analysis tells a convincing and well-organized story about the data and topic.
	10	A good balance between analytic narrative and illustrative extracts is provided.
**Written report**	11	Enough time has been allocated to complete all phases of the analysis adequately,<br>without rushing a phase or giving it a once-over-lightly.
	12	The assumptions about, and specific approach to, thematic analyses are clearly explicated.
	13	There is a good fit between what you claim you do, and what you show you have done i.e., described method, and reported analysis are consistent.
	14	The language and concepts used in the report are consistent with the epistemological position of the analysis.
	15	The researcher is positioned as active in the research process; themes do not just 'emerge'.

The checklist of criteria for good thematic analysis proposed by [31].
